# Re: “Recovery of Elective Facial Plastic Surgery in the Post-Coronavirus Disease 2019 Era: Recommendations from the European Academy of Facial Plastic Surgery Task Force” by Unadkat et al.

**DOI:** 10.1089/fpsam.2020.0276

**Published:** 2020-10-02

**Authors:** David Edward James Whitehead, David A. Lowe, Robert E. Weir

**Affiliations:** ^1^ Department of Otorhinolaryngology, James Cook University Hospital, Middlesbrough, United Kingdom.; ^2^ Lee Health, Department of Medicine, COVID Unit, Cape Coral, Florida, USA.

## Considerations for Elective Facial Plastic Surgery in the Post-Coronavirus Disease 2019 Era

The Rhinoplasty Society of Europe recently convened a webinar regarding this topic on May 3, 2020, but this was not acknowledged within the EAFPS Task Force publication.^1^

We read your article with interest and wish to note the following:

(1) The authors state their aim is to make recommendations and guidance involving no one but surgeons. We would argue that collaboration should involve specialties outside of surgery, for example, infectious diseases, virology, and public health.
(2) The authors^1^ refer to “very well-documented cases of widespread infection of operating-room personnel” yet without reference to such cases. Please note that similar claims have been rejected in other studies.^2^
(3) There is an omission in the recommendations regarding consent. This may form the basis of a legal challenge similar to the UK Supreme Court, Montgomery v Lanarkshire Health Board case. Concern that surgery may accelerate and exacerbate disease progression of COVID-19 is evident in a small but important study^3^ that showed 20.6% of asymptomatic patients who unwittingly had elective surgery during the incubation period of COVID-19 died. This cohort, however, may not be comparable with those undergoing elective facial plastic surgery (elFPS) as simpler surgery had more favorable outcomes.^3^ Although an ongoing global collaboration (CovidSurg) is informing decision making with 12,000 patients in 600 hospitals in 70 countries, this may not be authentic to the needs elFPS.
(4) Telemedicine is not uniformly feasible and prone to failures as evident with the Attend Anywhere platform within the U.K.'s NHS. The use of local anesthetic and office-based procedures is controversial and may enhance risk of infection transmission by bioaerosol.
(5) There is a clear need to mitigate for false negative COVID-19 patients and care workers in a cold COVID-19 site. Yet the use of povidone iodine (PVP-I) disinfectant is problematic and concerns as to its use are expressed in the literature of neurotoxicity.^4^ It may, in fact, augment the olfactory effects of COVID-19. Furthermore, tranexamic acid as a potential repurposed drug has not been approved in this role by any regulatory authority.
(6) Quality of surgery is important. The challenge will be to reduce exposure risk while not diminishing surgical technique. We foresee the increased use of plume extractors or novel extraoral suction devices such as DuraMax.
(7) Multidisciplinary collaboration and accurate data dissemination are vital at the prerecommendation stage. Proper consensus and accountability protect against legal challenge and unintended consequence.
(8) elFPS will coexist with COVID-19 for the foreseeable future providing increased evidential opportunities to understand actual outcomes. Global multidisciplinary collaboration is paramount together with opportunities to scrutinize and comment transparently on these data.
